# The congenital birth defects burden in children younger than 14 years of age, 1990 – 2019: An age-period-cohort analysis of the global burden of disease study

**DOI:** 10.7189/jogh.14.04012

**Published:** 2024-01-19

**Authors:** Xin-yu Li, Meng-jie Hou, Xiang-meng Kong, Jia-jie Lv, Cheng-hao Yang, Da-tao Li, Ru-hong Zhang

**Affiliations:** 1 Department of Plastic and Reconstructive Surgery, Shanghai Ninth People's Hospital, Shanghai Jiao Tong University School of Medicine, Shanghai, China; 2 Department of Cardiology, Shanghai Ninth People's Hospital, Shanghai Jiao Tong University School of Medicine, Shanghai, China; 3 Department of Vascular surgery, Shanghai Ninth People's Hospital, Shanghai Jiao Tong University School of Medicine, Shanghai, China; 4 Department of Vascular surgery, Putuo People’s Hospital, School of Medicine, Tongji University, Shanghai, China

## Abstract

**Background:**

This study aims to delineate the burden of congenital birth defects (CBDs) in children under 14 years of age from 1990 to 2019, using an age-period-cohort framework to analyse data from the Global Burden of Disease Study (GBD).

**Methods:**

Data on prevalence cases, age-standardised prevalence rates (ASPRs), death cases, and age-standardised death rates (ASDRs) of congenital birth defects (CBDs) from 1990 to 2019 were obtained from GBD 2019. Using this data set, we conducted an age-period-cohort (APC) analysis to examine patterns and trends in mortality, prevalence, and disability-adjusted life years (DALYs) associated with CBDs, while exploring correlations with age, time periods, and generational birth cohorts. Furthermore, to quantify the temporal trends, we calculated the estimated annual percentage changes (EAPCs) for these parameters.

**Results:**

The global prevalence of CBDs decreased from 1404.22 to 1301.66 per 100 000 with an EAPC of −0.18% from 1990 to 2019. CBD mortality decreased by 42.52% between 1990 and 2019, with the global age-standardised death rate declining from 49.72 to 25.58 per 100 000. The age-standardised DALY rate decreased from 4529.16 to 2393.61 per 100 000. Prevalence declined most notably among older children. The risk of CBDs reached its lowest during adolescence (10–14 years) across all regions. The most recent period (2015–2019) showed a reduced risk of prevalence compared to 2000–2004. Earlier birth cohorts displayed declining tendencies followed by slight increases in risk.

**Conclusions:**

This study demonstrates encouraging global reductions in the burden of CBDs among children over the past three decades. Prevalence, mortality, and DALYs attributable to CBDs have exhibited downward trajectories, although regional disparities remain. APC analysis provides valuable insights to inform prevention and management strategies for pediatric CBDs.

Congenital birth defects (CBDs) encompass a diverse range of structural, functional, and metabolic malformations present at birth, with significant implications for global child health [1]. CBDs are a leading cause of neonatal mortality and contribute to long-term disability, with varying degrees of severity affecting individual quality of life, family dynamics, and health care systems [2]. The etiological landscape of CBDs is multifaceted, including genetic, environmental, and maternal health factors, which can influence the occurrence and severity of these conditions [3]. Worldwide, it is estimated that CBDs affect approximately six percent of infants, leading to the demise of 240 000 newborns within the first 28 days of life and causing the death of an additional 170 000 children between the ages of one and 59 months annually [3]. In the year 2019, these defects emerged as the fourth leading cause of mortality among children under the age of five, comprising close to 10% of all fatalities in this age group [4]. However, comprehensive epidemiological profiling of congenital birth defects, especially regarding disease burden metrics like prevalence, mortality and disability-adjusted life years, remains lacking. Quantifying and analysing recent global and regional trends is critical for informing resource allocation and tailored prevention strategies to address this substantial yet under-examined child health challenge.

To bridge the existing gap in our understanding, the Global Burden of Disease (GBD) study – launched by the World Health Organization and the World Bank – provides an extensive epidemiological analysis detailing the mortality and morbidity attributable to major diseases, injuries, and their potential risk factors [5,6]. By harnessing the most recent GBD 2019 data set along with the age-period-cohort (APC) model, we elucidate the longitudinal trends in the burden of CBDs among children under 14 years of age spanning the last three decades. The insights gleaned from these trends will deepen our understanding of the factors contributing to the burden of CBDs in this young demographic. Furthermore, this knowledge will underpin the theoretical basis for tailoring and enhancing personalised intervention strategies which can be adapted for national health care systems and individual patient needs.

## METHODS

### Data source and definitions

This was a cross-sectional study that used data from the GBD 2019 in 204 countries and territories. Children who were aged 0 to 14 years were included in the analysis. Our study utilises data from the 2019 iteration of the GBD 2019, adhering closely to its established methodological framework and analytical strategies. The GBD 2019 study amassed epidemiological data across a consortium of 204 nations and territories, evaluating the prevalence of 369 diseases and injuries alongside 87 risk determinants. The intricate methodologies that form the foundation of the GBD 2019 have been extensively delineated in preceding scholarly works [7,8]. The GBD collaborative applies a methodical approach, harnessing comprehensive data sets spanning various age cohorts, temporal spans, geographic locales, and multifarious health-related factors. These data sets are meticulously analysed using harmonised analytical tools within a Bayesian inferential framework. Such a stratagem affords the advantage of leveraging extant data sets to surmount the barriers posed by the paucity of complete health care information access within the populace. Consequently, this facilitates a more informed quantification of the global disease burden, ensuring that estimations are inclusive of all constituent countries and territories [9–11].

The cataloging system of the GBD delineates a meticulous and non-overlapping compendium of health conditions, systematically classified into four echelons of hierarchy [12]. Within this classification, birth defects are enumerated under the primary rubric of non-communicable diseases at level 1, with a further specification under other non-communicable conditions at level 2. The subgroup identified as Congenital Birth Defects (B12.1) incorporates eleven conditions at level 4.

In adherence to ethical frameworks, the study was executed in concordance with the principles established by the Declaration of Helsinki. Utilising de-identified aggregated data sets obviated the need for individual informed consent, a stipulation that was reviewed and sanctioned by the Institutional Review Board of the University of Washington, which approved the waiver of consent for this research (https://www.healthdata.org/Data-tools-practices/data-practices/ihme-free-charge-non-commercial-user-agreement).

The objective of this investigation was to scrutinise the trends in prevalence, disability-adjusted life years (DALYs), and mortality across three decades, from 1990 to 2019. This analysis employed a suite of metrics, comprising the absolute number of deaths, incidence of prevalence, age-standardised prevalence rates, age-standardised mortality rates, and estimated annual percentage changes (EAPCs). These measures were directly procured from the GBD 2019. The 95% uncertainty intervals (UIs) were delineated by the 2.5th and 97.5th percentiles, representing the 25th and 975th values, respectively, from the ordered set of 1000 estimates. These intervals were computed in accordance with the algorithmic methodology employed by the GBD study.

To ensure consistency, the study population was divided into six age groups: 0–6 days, 7–27 days, 28–364 days,1–4 years,5–9 years and 10–14 years.

### State-level data

Moreover, the analysis integrated the Socio-demographic index (SDI) for each nation and region under study. The SDI is a synthetic metric that aggregates three core dimensions: per capita income, average educational attainment, and fertility rates among females younger than 25 years [13]. The SDI is quantified on a continuum from 0 to 1, where ascending values correlate with augmented socioeconomic status. Consequent to the computation of these values, we stratified all nations and regions into quintiles predicated on their SDI metrics for the year 2019, thereby facilitating a comparison across a spectrum of socio-demographic echelons, from high to low.

### Analysis of overall temporal trends in CBDs disorders in children

Between 1990 and 2019, we analysed the temporal trends in the prevalence of CBDs by employing raw counts and ASR. To ascertain the age-specific prevalence rates (ASPR) of CBDs in pediatric cohorts, we utilised direct age standardisation, operating under the premise that the rates conform to a weighted summation of independent Poisson random variables, as detailed in our referenced methodologies [14,15]. Moreover, we conducted an examination of the proportional distribution of CBDs prevalence in children, dividing the subjects into three distinct age groups: under five years, five–nine years, and 10–14 years. This categorisation facilitated an illustration of the temporal changes in the age-specific prevalence of CBDs.

### Age-Period-Cohort analysis of CBDs disorders in children

In this study, we utilised the Age-Period-Cohort (APC) model framework, an advanced statistical tool heralded for its applications beyond traditional analysis in health and social sciences [16]. This model was employed to dissect the temporal dynamics of prevalence with a particular focus on age, period, and birth cohort [16,17]. The APC model has been extensively applied in epidemiological investigations of non-communicable diseases, including congenital heart disease, as evidenced by prior research [17,18]. Despite its utility, the model is not without its challenges; a notable issue is the 'identification problem' which arises due to the exact collinearity among the three variables, as age is the difference between period and birth cohort, rendering the independent effects of each variable statistically indistinguishable [16]. To address this conundrum, our approach involved generating estimable parameters and functions of the APC model without resorting to the imposition of arbitrary constraints [18,19]. The intricate methodological nuances of the APC model and its implementation have been expansively explicated in the existing literature [20]. In our study, we harnessed population and disease burden data for CBDs to feed the APC model. To maintain methodological rigor, the APC model necessitates that the intervals for age be congruent with those of the period; thus, we used 5–year age groups aligned with 5–year calendar periods for consistency. Given that the childhood age span was delineated as 0–14 years, we segmented this into three age groups: <5 years, five–nine years, and 10–14 years, for subsequent analysis. The temporal scope of the study, spanning from 1990 to 2019, was accordingly divided into six quinquennial periods: 1990–1994, 1995–1999, 2000–2004, 2005–2009, 2010–2014, and 2015–2019. To complement this, we delineated 12 partially overlapping decennial birth cohorts: 1940–1949, 1945–1954, 1950–1959, 1955–1964, 1960–1969, 1965–1974, 1970–1979, 1975–1984, 1980–1989, 1985–1994, 1990–1999, and 1995–2004. This stratification allowed for a detailed examination of the influences exerted by age, period, and cohort on the trends in congenital birth defects.

The APC model offers a nuanced approach to analysing disease prevalence, providing estimates on both the overarching temporal trend and age-specific trends within the prevalence data. The general temporal trend, or the net drift, is quantified as the annual percentage change of prevalence, encapsulating the aggregated effects attributable to calendar time and consecutive birth cohorts. Conversely, the age-specific temporal trend, referred to as the local drift, reflects the annual percentage change within each age group.

A seemingly minor drift value, expressed as a percentage change per year, can translate to a significant alteration in the modeled prevalence rate over a three-decade span. To test the statistical significance of these trends, a Wald χ^2^ test was employed. Within the confines of the APC model, age effects are manifested through the modeled longitudinal age-specific rates across various birth cohorts, which are subsequently adjusted for deviations arising from different periods. Conversely, the period and cohort effects are elucidated as relative risks of prevalence, calculated as the ratio of age-specific rates for each period or cohort against a designated reference. The selection of this reference period or cohort is arbitrary and does not influence the interpretability of the results. To forecast the disease burden from 1990 to 2045, we employed a log-linear age-period-cohort model. This approach limits linear trend projection and controls exponential growth, making it appropriate for adapting to current trends. The model was executed in R, utilising the NORDPRED package [21].

The entirety of the analyses, as well as the visual representations of the data, were conducted using R software, version 4.2.1.

## RESULTS

### Prevalence

**Figure 1:**
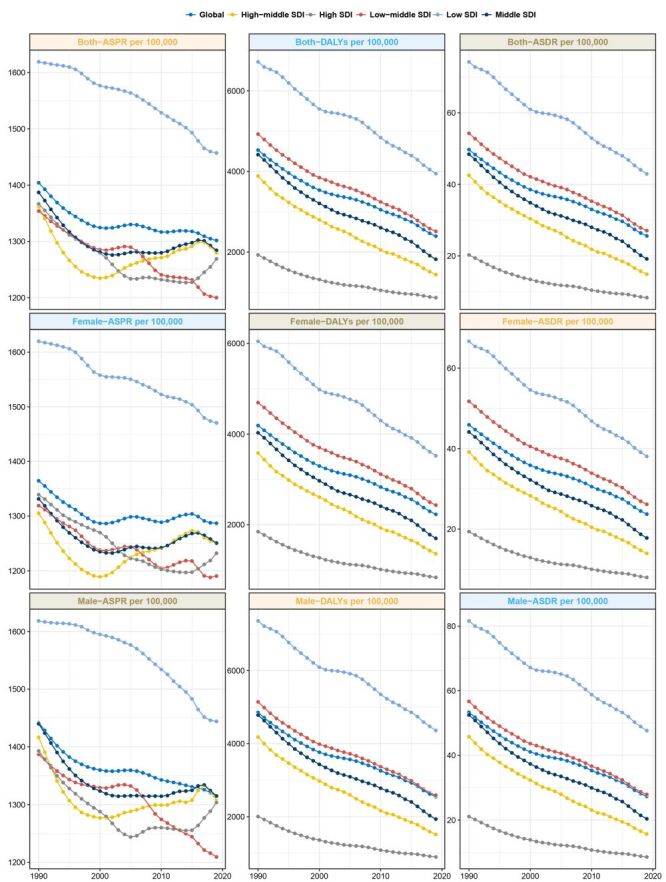
Trends in congenital birth defects prevalence, deaths and disability-adjusted life-years from 1990 to 2019.

**Figure 2:**
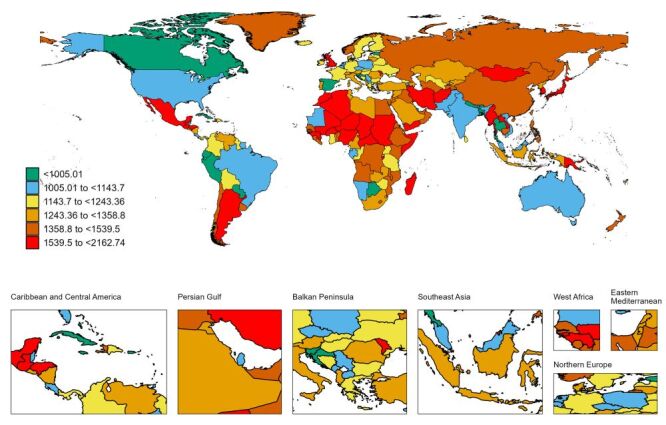
The global disease burden of congenital birth defects prevalence for both sexes in 204 countries and territories.

The global prevalence of CBDs was estimated at 255 090.41 cases (95% UI = 224 268.68–289 785.27) in 2019 (**Table 1**, **Figure 1**). The global ASPR of CBDs has declined from 1404.22 per 100 000 population to 1301.66 per 100 000 population in three decades, with an EAPC of −0.18 (95% confidence interval (CI) = −0.22 to −0.14, **Table 1**). Globally, the incidence of CBDs is higher in males than in females, with the ratio between males and females reaching its maximum at ages 10–14 (Figure S1 in the **Online Supplementary Document**). ASPR decreased in all SDI regions, with the lowest (1200.14 per 100 000) in the low-middle SDI region and the highest in the low SDI region (1457.05 per 100 000) (**Table 1**, **Figure 1**). Across the five SDI's, the incidence was higher in men than women in most age groups (except for low SDI, Figure S1 in the **Online Supplementary Document**). Among the 21 regions, high-income Asia Pacific (1847.11 per 100 000) and Oceania (1655.95 per 100 000) had significantly higher ASPR than the others (**Table 1**, **Figure 2**).

**Table1:** Prevalence of congenital birth defects between 1990 and 2019 in 0 to 14 y at the global and regional level

	1990		2019		EAPC (95% CI)
	**No (95% UI)**	**ASR**	**No (95% UI)**	**ASR**	
Overall	246 295.52 (215 257.15–282 139.56)	1404.22 (1227.26–1608.58)	255 090.41 (224 268.68–289 785.27)	1301.66 (1144.38–1478.7)	−0.18 (−0.22 to −0.14)
Female	116 426.82 (102 278.76–132 691.7)	1364.47 (1198.66–1555.08)	121 995.89 (107 441.14–138 327.91)	1286.81 (1133.28–1459.08)	−0.12 (−0.17 to −0.07)
Male	129 868.70 (112 902.2–149 408.13)	1441.88 (1253.51–1658.82)	133 094.52 (117 065.29–151 384.06)	1315.58 (1157.14–1496.36)	−0.24 (−0.27 to −0.2)
High SDI	23 598.47 (20 907.72–26 718.56)	1366.63 (1210.81–1547.32)	20 717.66 (18 448.72–23 202.99)	1268.98 (1130.01–1421.21)	−0.32 (−0.39 to −0.24)
High-middle SDI	41 352.26 (36 390.61–46 954.16)	1362.33 (1198.87–1546.88)	31 380.59 (27 879.64–35 310.89)	1279.70 (1136.93–1439.98)	−0.02 (−0.12 to 0.09)
Middle SDI	80 640.62 (70 190.49–92 906.97)	1387.11 (1207.36–1598.11)	71 089.02 (62 567.84–81021.79)	1284.12 (1130.2–1463.55)	−0.15 (−0.23 to −0.07)
Low-middle SDI	61 388.39 (53 342.16–71 162.9)	1353.96 (1176.5–1569.55)	62 833.18 (55 461.06–71 796.33)	1200.14 (1059.33–1371.34)	−0.37 (−0.4 to −0.34)
Low SDI	39 183.06 (33 538.19–45 708.12)	1619.03 (1385.78–1888.64)	68 900.39 (59 388.84–79 827.77)	1457.05 (1255.91–1688.13)	−0.37 (−0.39 to −0.34)
Andean Latin America	1375.27 (1178.54–1626.17)	914.14 (783.37–1080.91)	1652.37 (1418.71–1937.1)	913.37 (784.21–1070.76)	−0.03 (−0.09 to 0.04)
Australasia	518.89 (450.71–595.54)	1131.21 (982.57–1298.31)	627.93 (549–723.47)	1145.13 (1001.19–1319.35)	0.05 (−0.02 to 0.12)
Caribbean	1214.59 (1035.89–1416.42)	1064.17 (907.6–1241)	1385.54 (1185.72–1612.59)	1185.88 (1014.85–1380.21)	0.38 (0.33 to 0.44)
Central Asia	3293.93 (2834.29–3795.68)	1319.90 (1135.72–1520.95)	3505.68 (3017.38–4067.93)	1303.24 (1121.71–1512.25)	0.25 (0.06 to 0.45)
Central Europe	3742.15 (3276.39–4260.23)	1292.78 (1131.87–1471.76)	2033.21 (1778.09–2303.94)	1152.84 (1008.19–1306.35)	−0.1 (−0.25 to 0.06)
Central Latin America	10 179.31 (8562.43–12147.42)	1588.10 (1335.85–1895.15)	9871.14 (8306.57–11 677.13)	1509.62 (1270.35–1785.81)	−0.32 (−0.42 to −0.21)
Central sub-Saharan Africa	4239.41 (3614.24–4974.14)	1641.08 (1399.08–1925.5)	8161.73 (6956.77–9538.35)	1430.61 (1219.4–1671.91)	−0.34 (−0.43 to −0.26)
East Asia	47 420.51 (40 813.07–55 108.77)	1415.79 (1218.52–1645.34)	32 750.09 (28 834.78–37 126.12)	1407.36 (1239.11–1595.41)	0.12 (−0.05 to 0.29)
Eastern Europe	7632.25 (6641.12–8699.31)	1483.44 (1290.8–1690.83)	4915.13 (4301.94–5591.65)	1326.29 (1160.83–1508.84)	0.23 (0.01 to 0.46)
Eastern sub-Saharan Africa	15 332.36 (12 813.92–18 152.02)	1696.67 (1417.98–2008.69)	25 030.54 (21 222.51–29 403.21)	1417.92 (1202.2–1665.62)	−0.63 (−0.66 to −0.6)
High-income Asia Pacific	6676.81 (5692.95–7898.27)	1894.53 (1615.36–2241.12)	4311.61 (3677.38–5053.04)	1847.11 (1575.41–2164.75)	−0.09 (−0.14 to −0.04)
High-income North America	7157.58 (6334.08–8033.46)	1165.52 (1031.41–1308.12)	7182.60 (6380.11–8017.12)	1083.83 (962.73–1209.75)	−0.48 (−0.61 to −0.35)
North Africa and Middle East	23 685.56 (20 507.39–27 680.94)	1648.01 (1426.88–1926)	26 944.25 (23 456.2–31 224.38)	1532.29 (1333.93–1775.7)	−0.20 (−0.25 to −0.15)
Oceania	405.79 (339.93–479.84)	1544.29 (1293.67–1826.1)	799.82 (674.88–946.93)	1655.95 (1397.28–1960.53)	0.34 (0.29 to 0.39)
South Asia	51 812.50 (45 212.35–59 928.65)	1180.29 (1029.94–1365.18)	52 418.90 (46 487.97–59 637.18)	1014.38 (899.6–1154.06)	−0.45 (−0.5 to −0.39)
Southeast Asia	25 701.31 (21 543.05–30 690.54)	1492.27 (1250.83–1781.95)	22 338 (19 144.49–26 231.22)	1323.55 (1134.33–1554.23)	−0.30 (−0.34 to −0.26)
Southern Latin America	2178.11 (1873.07–2548.59)	1458.89 (1254.58–1707.04)	2274.25 (1961.96–2652.26)	1525.72 (1316.22–1779.32)	0.35 (0.28 to 0.42)
Southern sub-Saharan Africa	2834.65 (2320.04–3455.57)	1391.22 (1138.66–1695.96)	2995.89 (2515.18–3630.09)	1268.68 (1065.11–1537.24)	0.15 (−0.01 to 0.31)
Tropical Latin America	5947.90 (5122.72–6917.85)	1102.59 (949.63–1282.4)	5031.89 (4355.96–5851.68)	1012.19 (876.23–1177.1)	−0.36 (−0.46 to −0.27)
Western Europe	9268.05 (8260.87–10 410.91)	1304.41 (1162.66–1465.26)	8633.03 (7720.24–9665.19)	1254.41 (1121.77–1404.38)	−0.15 (−0.21 to −0.08)
Western sub-Saharan Africa	15 678.59 (13 207.6–18 673.26)	1785.37 (1503.99–2126.38)	32 226.84 (27 350.04–38 203.79)	1624.90 (1379.01–1926.27)	−0.34 (−0.4 to −0.28)

ASR – age-standardised rate, CI – confidence interval, EAPC – estimated annual percentage change, SDI – sociodemographic index, UI – uncertainty interval

### Mortality

In 2019, the number of deaths attributed to CBDs reached 5013.33 (95% UI = 3986.27–6491.41), marking a 42.52% decrease from the 1990 count of 8721.54 (95% UI = 5904.33–12 972.58) as shown in Table S1 in the **Online Supplementary Document**. The ASDR of the five SDI regions decreased with time. The ASDR of the High SDI was the lowest (8.33 per 100 000), while the ASDR of the low SDI was the highest (42.93 per 100 000) (Table S1 in the **Online Supplementary Document**). In the five SDI, the ratio of male to female ASDR showed a decreasing trend with the increase of age (Figure S2 in the **Online Supplementary Document**). In 30 years, the 2019 ASDR in 21 regions was lower than the 1990 ASDR. Western sub-Saharan Africa had the highest ASRD in 2019 (51.93 per 100 000) (Figure S3A in the **Online Supplementary Document**).

### DALYs

In 2019, the number of DALYs attributed to CBDs reached 469 083.64 (95% UI = 379 015.44–601 305.28) as shown in Table S2 in the **Online Supplementary Document**. This was accompanied by an EAPC of −1.93 (95% CI = −2.02 to −1.84) as reflected in Table S2 in the **Online Supplementary Document**. Notably, the male-female DALY ratio declined with age, suggesting that CBDs impose a higher burden on female children older than one year (Figure S4 in the **Online Supplementary Documen**t). The age-standardised DALY rate of the five SDI regions decreased with time. The EAPC of High-Middle SDI was the largest (−3.13; 95% CI = −3.21 to −3.05), the age-standardised DALY rate of High SDI was the lowest, and the age-standardised DALY rate of Low SDI was the highest (**Figure 1**, Figure S3B and Table S2 in the **Online Supplementary Document**).

### Temporal trends in CBDs burden in children across age groups

**Figure 3:**
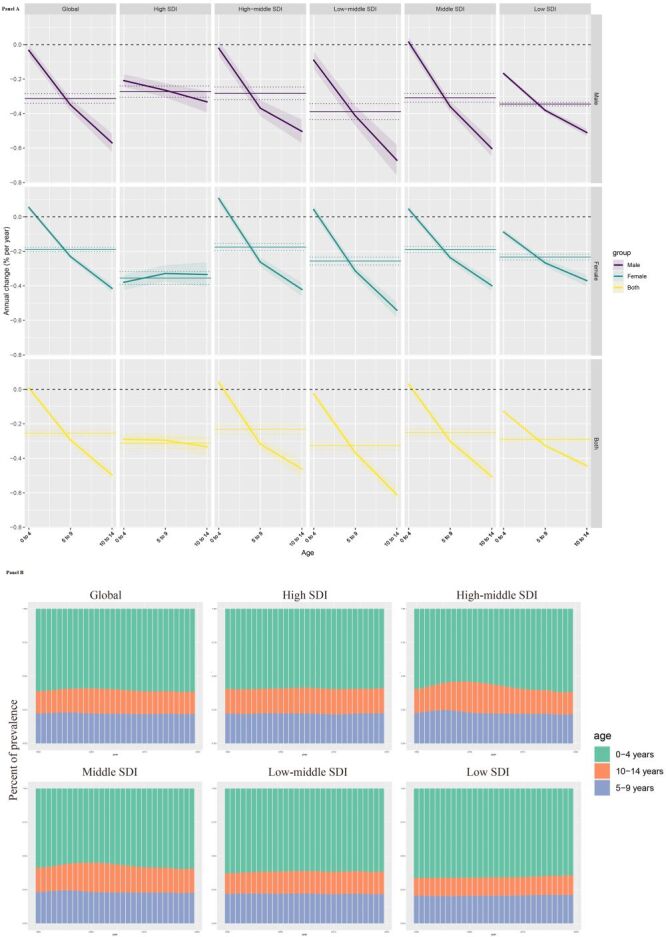
Local drift and age distribution of prevalence from 1990 to 2019 for congenital birth defects disorders across SDI quintiles. **Panel A.** Local drift of prevalence from 1990 to 2019 for congenital birth defects disorders for 3 age groups. The dots and shaded areas denote the local drift (i.e. annual percentage change of age-specific prevalence, % per year) and their corresponding 95% confidence intervals. **Panel B**. Temporal changes in age distribution of congenital birth defects disorders prevalence from 1990 to 2019.

We quantified the annual changes in the prevalence of CBD disorders across various age cohorts. Utilising the APC model, we elucidated the local drifts in prevalence (**Figure 3**, panel A and Table S3 in the **Online Supplementary Document**). Our analysis reveals a discernible decrease in the prevalence of CBD disorders among children, particularly evident when comparing the five–nine-year age group with the 10–14-year cohort. This declining trend becomes more pronounced as children age, especially from the five–nine to the 10–14-year groups. Notably, the smallest decrease was observed in the 10–14-year cohort. We observed that most SDI regions show a downward trend. In female children with High SDI, aged 0–14 years, the trend of local drift is manifested first by rising and then falling (all values are less than 0) (**Figure 3**, panel A).

Temporal changes in the age distribution of CBD disorders prevalence in children were illustrated in **Figure 3**, panel B. We found that globally and in all five SDI regions, the proportion of CBD disorders prevalence in children aged 0–4 years remained dominant over the 30-year period.

### Age, period and birth cohort effects on CBDs disorders prevalence in children

**Figure 4:**
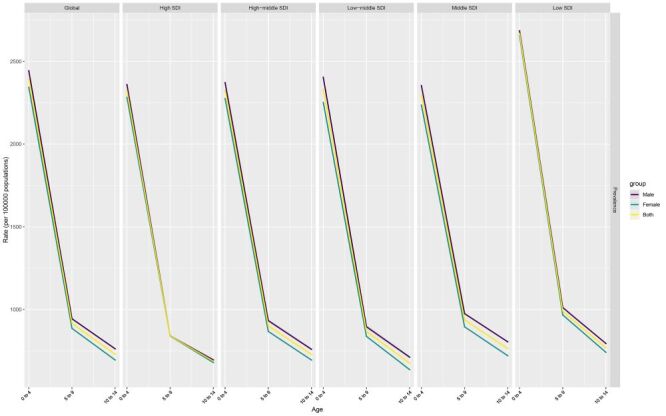
Age effects are illustrated by the fitted longitudinal age-specific rates for a given number of birth cohorts adjusted for period deviations. The dots and shaded areas denote the prevalence rates or rate ratios and their corresponding 95% confidence intervals.

**Figure 5:**
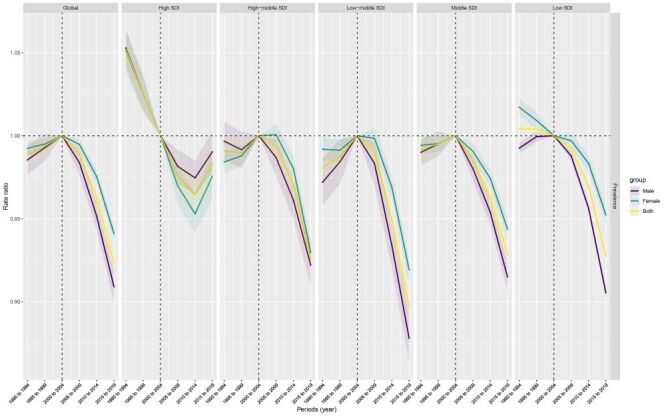
Period effects are illustrated by the period relative risk of prevalence (prevalence rate ratio) and calculated as the ratio of age-specific rates from 1990–1994 period to 2015–2019 period, with the reference period set at 2000–2004. The dots and shaded areas denote the prevalence rates or rate ratios and their corresponding 95% confidence intervals.

**Figure 6:**
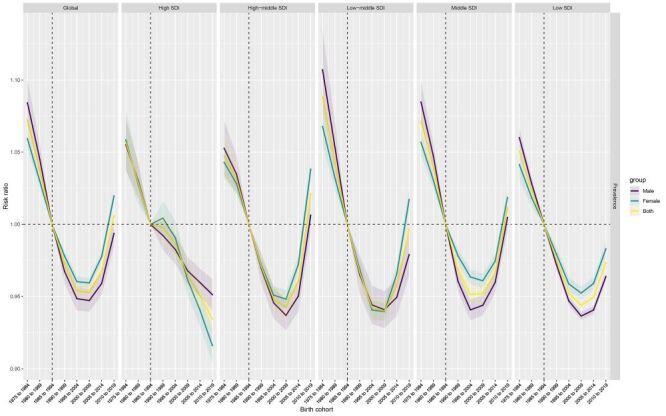
Birth cohort effects are illustrated by the cohort relative risk of prevalence (prevalence rate ratio) and calculated as the ratio of age-specific rates from 1975–1984 cohort to 2010–2019 cohort, with the reference cohort set at 1985–1994. The dots and shaded areas denote the prevalence rates or rate ratios and their corresponding 95% confidence intervals.

The age, period, and birth cohort effects derived from the APC model are illustrated in **Figure 4**, **Figure 5**, and **Figure 6**, and detailed in the online Tables S4-6 in the **Online Supplementary Document**. A consistent pattern emerged in the age effects across regions stratified by SDI, indicating that the nadir of risk was observed during the adolescent phase, specifically at ages 10–14 years, with a subsequent decrease in risk as age advanced. The high-middle SDI region, middle SDI region, and low-middle SDI generally had lower period risks over the study period. When assessing the period effects, individuals in the 2015–2019 period exhibited a decrease in risk compared to those in the baseline period of 2000–2004; this decrease ranged from a relative risk of 0.98 (95% CI = 0.97–0.99) in regions with high SDI, to 0.93 (95% CI = 0.92–0.94) in both high-middle and middle SDI regions.

Analysis of birth cohort effects revealed a global pattern characterised by an initial decline followed by an increased risk of disorder prevalence in successive birth cohorts. This trend was largely mirrored across most regions classified by SDI, with high SDI regions exhibiting particularly reduced risks in prevalence among successive birth cohorts. When comparing individuals born in the 1995–2004 reference cohort to those born in the 2010–2019 cohort, the relative cohort risk increased slightly, ranging from 1.02 (95% CI = 1.01–1.03) in the high-middle SDI region to 1.01 (95% CI = 1.00–1.02) in the middle SDI region

### Future burden of CBDs

Figure S5 in the **Online Supplementary Document** displays the forecasted trends in DALYs for global and five SDI categories related to CBD. The projection indicates a global decline in DALYs for children under 14 years affected by CBD. This downward trend is mirrored in countries categorised as high-middle, low-middle, and middle SDI. In contrast, countries with a low SDI demonstrate a consistent upward trend in DALYs associated with CBD.

## DISCUSSION

CBDs represent the predominant etiology of neonatal mortality, with an estimated 10–20% of such fatalities being attributable to these anomalies [22,23]. Accurate and comprehensive monitoring of the burden or incidence of CBDs is an essential part of achieving the Millennium Goal of reducing infant mortality and the 2030 Sustainable Development Goals [24]. To our knowledge, this is the first use of APC models to analyse time trends in CBD on a global scale, allowing comparisons between different regions.

Globally, our results demonstrate an overall decline in the ASPR of CBDs, from 1404.22 to 1301.66 per 100 000 population between 1990 and 2019. This downward trajectory was mirrored across all SDI regions, albeit with variances in the magnitude of the decrement. However, notable disparities remain, as underscored by the substantially higher ASPR observed in 2019 among the low SDI region (1457.05 per 100 000) compared to high SDI areas (1268.98 per 100 000). Closing this gap by enhancing health care provisions and resources for underprivileged populations must constitute a pivotal public health priority. The observed disparities likely stem from a range of inequalities spanning from preconception to postnatal care. Factors contributing to these disparities include limited access to prenatal screening and diagnostic services, variations in the quality of antenatal care, differing sociocultural perceptions of disability, a lack of safe delivery facilities, and inadequate rehabilitative services in less advantaged areas [17,25]. The disparities in global health care are exacerbated by unequal access to ultrasound technology, variability in perinatal diagnostics, and diverse approaches to pregnancy and postnatal care [26]. Recognising these discrepancies across different regions and populations is vital for understanding and addressing global health disparities. Of course, due to the inherent limitations of the study's observational design, caution is required in establishing causality, and the results may not be universally applicable due to differences in data reliability and availability across regions. Severe birth anomalies are often identified with reliability, yet milder conditions frequently remain undiagnosed and untreated, especially in disadvantaged areas. To tackle these multifaceted issues, an integrated approach encompassing health care provision, community mobilisation, and policy innovation is essential. Key interventions should focus on ensuring equitable access to comprehensive preconception counselling, effective antenatal screening, safe childbirth practices, quality rehabilitative services, and robust social support networks [27]. A deep understanding of the specific barriers and enablers unique to each community is critical for devising and implementing context-sensitive strategies. Collaborative, action-oriented research, coupled with strong global partnerships, is imperative to catalyse meaningful progress in mitigating these health disparities [26].

Beyond prevalence, our analysis demonstrates an encouraging 42.52% reduction in CBD-related mortality between 1990 and 2019, with the global ASDR decreasing from 49.72 to 25.58 per 100 000 over this period. This trend of ameliorating ASDRs was reflected across all SDI regions classified by socioeconomic development. The disease burden imposed by CBDs, as quantified through DALYs, demonstrated consonant trends. Plausible drivers include medical advancements enabling improved survival for children born with congenital disorders, coupled with greater accessibility to these interventions over time [28]. However, notable disparities remain, as underscored by the substantially higher ASDR observed in 2019 among the low SDI region (42.93 per 100 000) compared to high SDI areas (8.33 per 100 000). Closing this gap by enhancing health care provisions and resources for underprivileged populations must constitute a pivotal public health priority [17].

APC model analysis allowed granular insights into the temporal dynamics shaping CBD burden across stratified age groups. Local drift values, reflecting the annual percent change in age-specific prevalence, pointed to a progressive decline in the prevalence of CBDs with ascending age. This reduction was most pronounced when comparing the 10–14-year age bracket against the preceding five–nine-year category. The mitigation of prevalence among older children may be indicative of reduced survival over time. Segmenting the pediatric population by age revealed that the proportion of CBD cases within the 0–4 year demographic remained consistently dominant over the decades, underpinning the necessity of interventions targeting this critical developmental window.

The independent effects of age, period, and cohort on CBD prevalence could be extracted through the APC framework. A coherent age effect was discernible, with the nadir of risk manifesting during adolescence between 10–14 years. This pattern held true universally across all SDI regions, resonating with the age-specific prevalence trends observed. Regarding period effects, the most recent time interval from 2015–2019 showed a modest but significant reduction in prevalence risk relative to the 2000–2004 reference period. This aligns with the overall declining trend in CBD rates over time. Finally, analysis of birth cohort influences indicated that earlier cohorts exhibited a declining tendency, followed by a slight reversal with increased risk among more recent generations. This parabolic pattern was observed to varying degrees across SDI regions. The low magnitude of risk ratios underscores the complex interplay of demographic and generational factors impacting secular shifts in the prevalence of CBDs.

The study has several limitations. Our analysis primarily relies on the GBD study framework, which compiles secondary data sources rather than original, primary data sets. This approach is particularly relevant in many countries in the Global South, including several African nations affected by conflict and social unrest, where data often depend on unvalidated estimations. These regions face numerous challenges, such as limited resources, quality of the diagnostics, expertise gaps, and inadequate infrastructure, which are essential for comprehensive epidemiological surveillance of CBDs. Such factors can introduce biases and potential inaccuracies into our findings. Additionally, GBD database does not provide data categorised by disease severity. This limitation restricts our ability to conduct an analysis comparing disease rates of similar severity across various countries. Furthermore, the observational design of our study inherently limits the ability to establish causative relationships between observed trends in prevalence, mortality, and disease burden, and their underlying determinants.

## CONCLUSIONS

Our analysis of the extensive GBD 2019 data set reveals encouraging downward trajectories in CBD prevalence, mortality, and DALYs over the past three decades. Nevertheless, notable disparities persist across geographic regions and socioeconomic strata. The prevalence of CBDs progressively declines with age, likely reflecting reduced survival among affected children over time. Our findings can inform resource allocation, public health planning, and targeted interventions to advance the prevention and management of CBDs globally.

## Additional material


Online Supplementary Document
