# Supplementary material for: The congenital birth defects burden in children younger than 14 years of age, 1990 – 2019: An age-period-cohort analysis of the global burden of disease study
Source: J Glob Health. 2024 Jan 19;14:04012. doi: 10.7189/jogh.14.04012 (PMC10802974; doi:10.7189/jogh.14.04012)
Supplement: Online Supplementary Document [file jogh-14-04012-s001.pdf]

Fig.S1: Ratio of male to female prevalence of congenital birth defects disorders in different age subgroups.

Fig.S2: Ratio of male to female deathes of congenital birth defects disorders in different age subgroups.

Fig.S3: Trends in congenital birth defects deathes and disability-adjusted life-years from 1990 to 2019. (A): Deathes rate for congenital birth defect; (B): Disability-adjusted life-years rate for congenital birth defect

Fig.S4: Ratio of male to female Disability-Adjusted Life-Years of congenital birth defects disorders in different age subgroups.

Fig.S5: Future Forecasts of Disability-Adjusted Life-Years for Congenital Birth Defects.

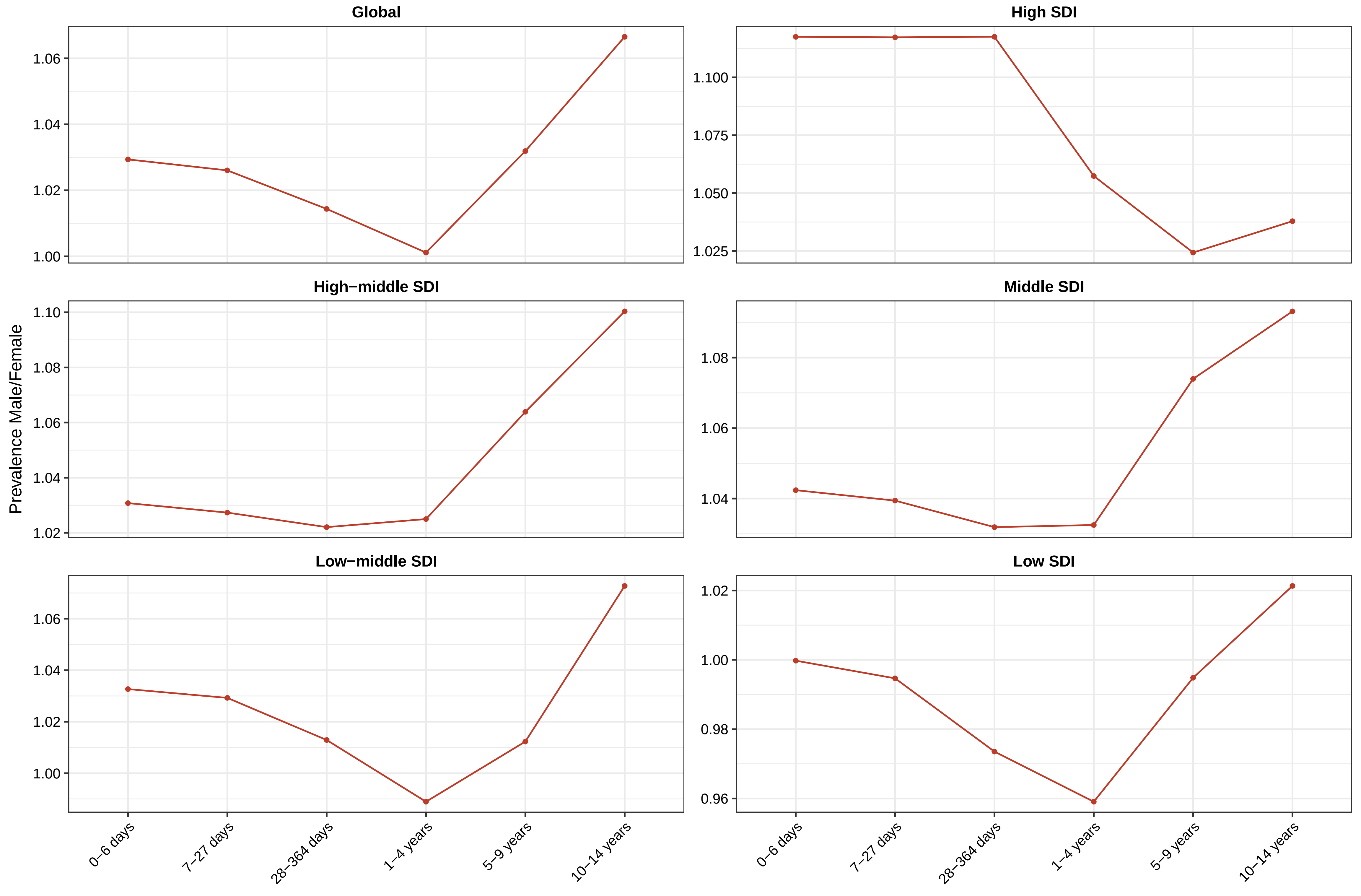
Global
High SDI
High−middle SDI
Middle SDI
Low−middle SDI
Low SDI
Prevalence Male/Female
1.00
1.02
1.04
1.06
1.025
1.050
1.075
1.100
1.08
1.10
0.96
0.98
0–6 days
7–27 days
28–364 days
1–4 years
5–9 years
10–14 years

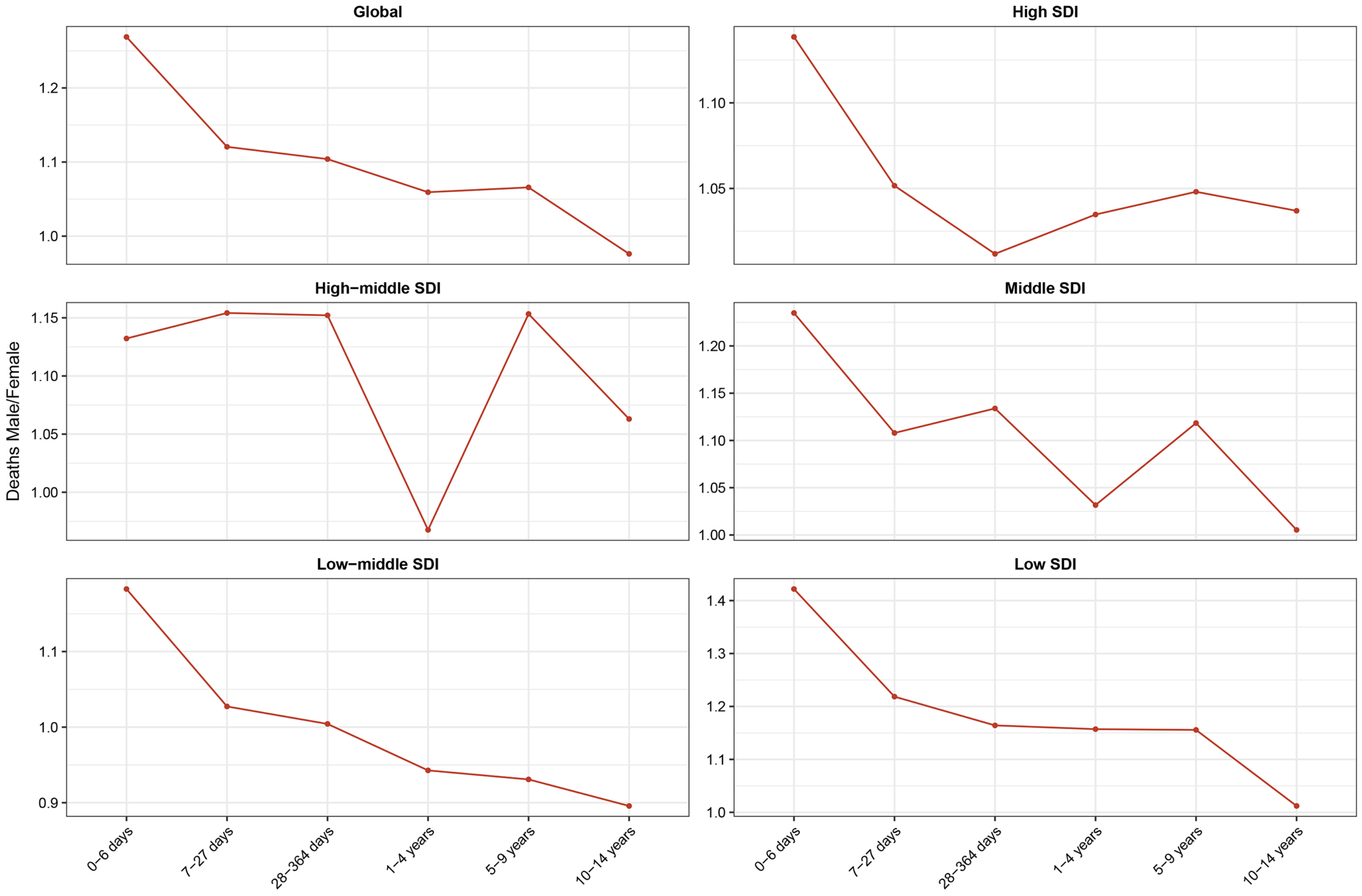
Global
High SDI
High−middle SDI
Middle SDI
Low−middle SDI
Low SDI
Deaths Male/Female
0−6 days
7−27 days
28−364 days
1−4 years
5−9 years
10−14 years

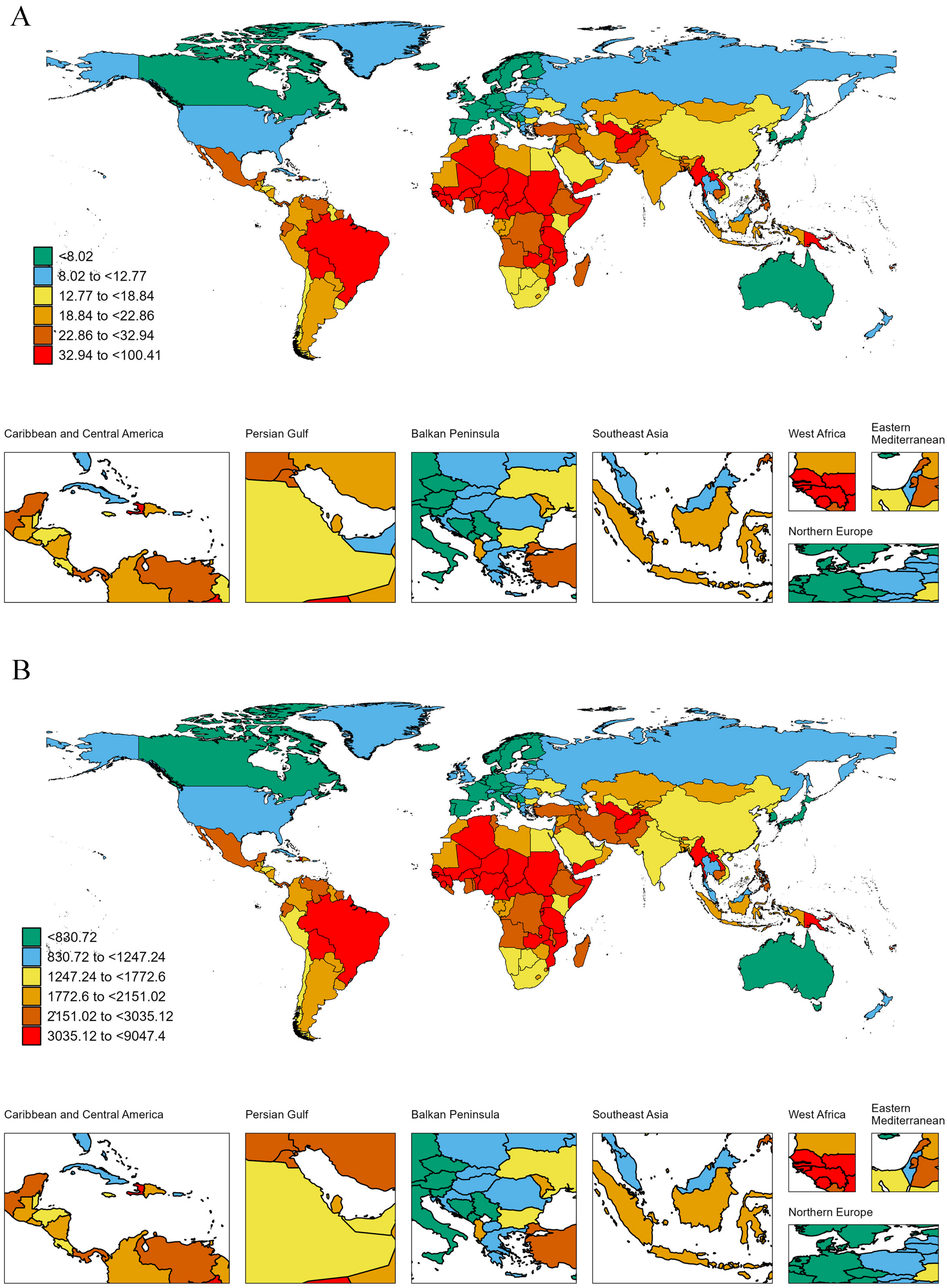
A
<8.02
8.02 to <12.77
12.77 to <18.84
18.84 to <22.86
22.86 to <32.94
32.94 to <100.41
Caribbean and Central America
Persian Gulf
Balkan Peninsula
Southeast Asia
West Africa
Eastern Mediterranean
Northern Europe
B
<830.72
830.72 to <1247.24
1247.24 to <1772.6
1772.6 to <2151.02
2151.02 to <3035.12
3035.12 to <9047.4
Caribbean and Central America
Persian Gulf
Balkan Peninsula
Southeast Asia
West Africa
Eastern Mediterranean
Northern Europe

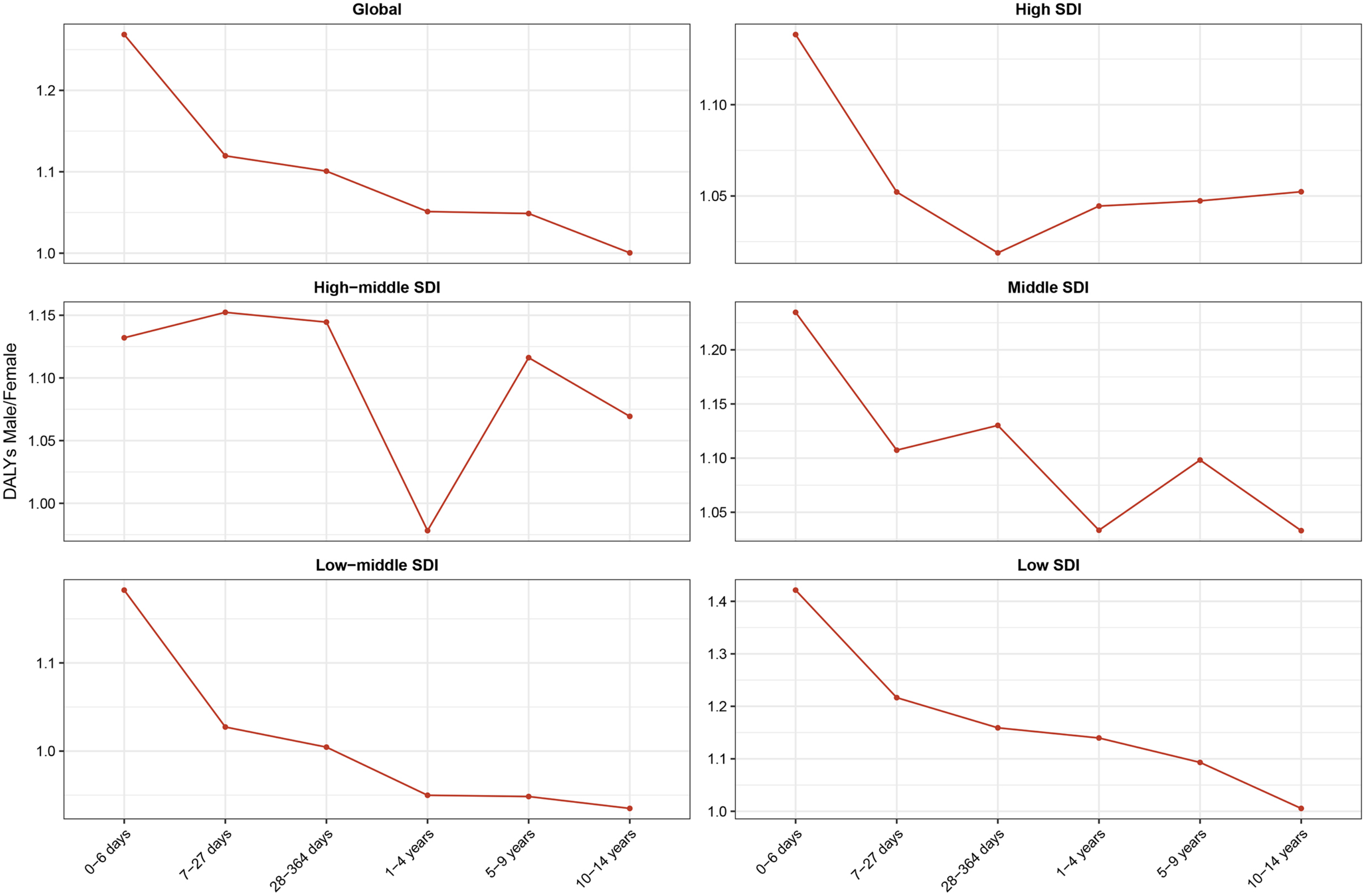

Global
High SDI
High−middle SDI
Middle SDI
Low−middle SDI
Low SDI
DALYs Male/Female
1.2
1.1
1.0
1.10
1.05
1.15
1.00
1.20
1.4
1.3
0−6 days
7−27 days
28−364 days
1−4 years
5−9 years
10−14 years

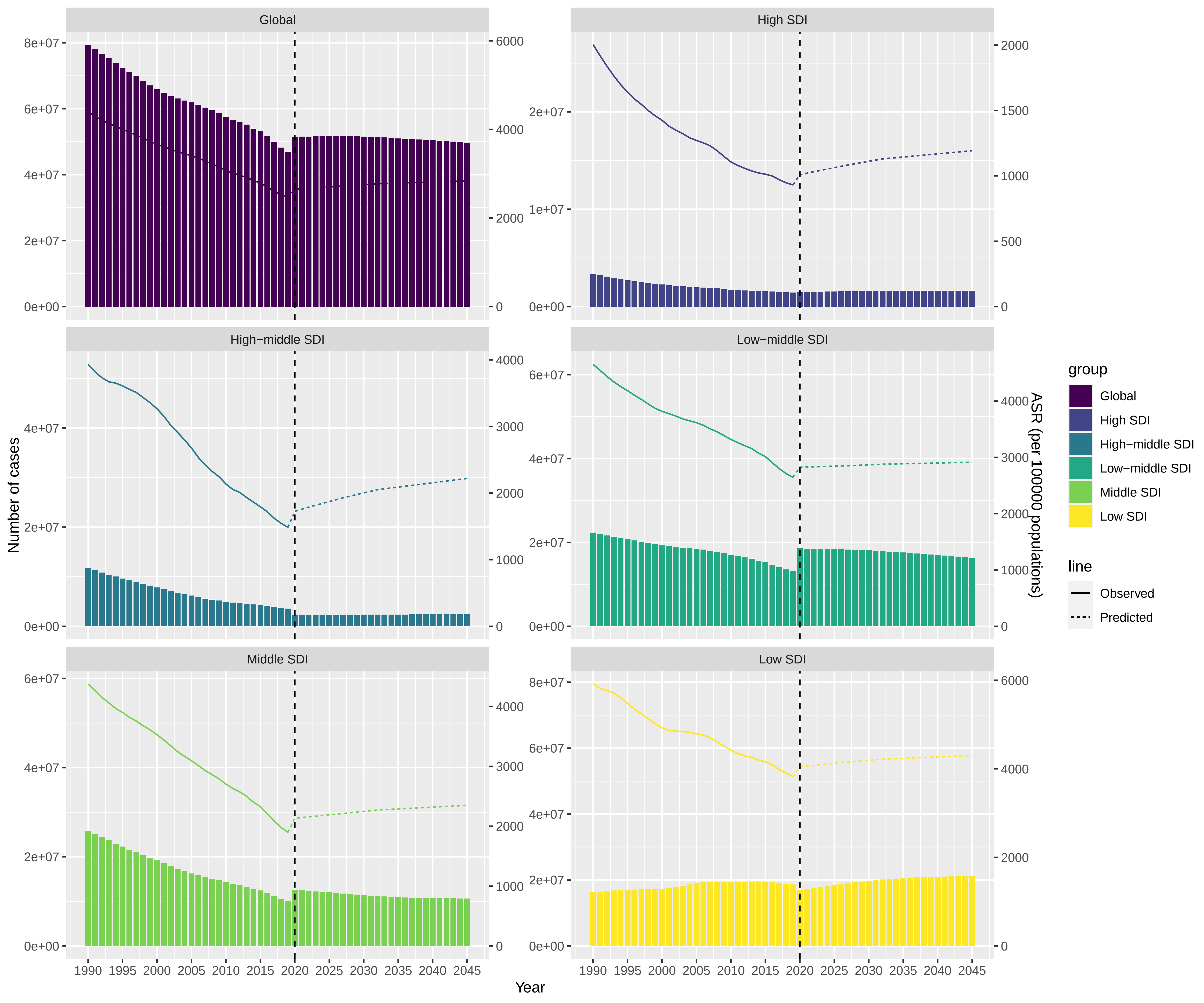
Global
High SDI
High−middle SDI
Low−middle SDI
Middle SDI
Low SDI
Number of cases
ASR (per 100000 populations)
Year
group
Global
High SDI
High−middle SDI
Low−middle SDI
Middle SDI
Low SDI
line
Observed
Predicted

TableS1: Deathes of congenital birth defects between 1990 and 2019 in 0 to 14 years at the global and regional level

	1990		2019		EAPC_95%CI
Location	Number_95%UI	ASR	Number_95%UI	ASR	
Overall	8721.54 [5904.33-12972.58]	49.72 [33.66-73.96]	5013.33 [3986.27-6491.41]	25.58 [20.34-33.12]	-2.01 [-2.11 to -1.91]
Female	3919.43 [2651.43-6154.83]	45.93 [31.07-72.13]	2249.24 [1776.66-2926.74]	23.72 [18.74-30.87]	-2.02 [-2.11 to -1.93]
Male	4802.11 [3068.93-7775.11]	53.32 [34.07-86.32]	2764.09 [2137.31-3727.12]	27.32 [21.13-36.84]	-2.01 [-2.11 to -1.9]
High SDI	349.79 [321.71-389.51]	20.26 [18.63-22.56]	135.96 [119.63-162.08]	8.33 [7.33-9.93]	-2.83 [-2.97 to -2.68]
High-middle SDI	1291.34 [1036.8-1560.86]	42.54 [34.16-51.42]	365.2 [307.27-432.26]	14.89 [12.53-17.63]	-3.31 [-3.41 to -3.22]
Middle SDI	2816.1 [2097.08-4068.41]	48.44 [36.07-69.98]	1060.28 [861.36-1306.36]	19.15 [15.56-23.6]	-2.78 [-2.92 to -2.64]
Low-middle SDI	2461.25 [1597.51-3644.9]	54.28 [35.23-80.39]	1417.26 [1161.16-1747.7]	27.07 [22.18-33.38]	-2.15 [-2.24 to -2.06]
Low SDI	1797.84 [739.39-3309.95]	74.29 [30.55-136.77]	2030.25 [1185.92-3207.79]	42.93 [25.08-67.84]	-1.77 [-1.85 to -1.69]
Andean Latin America	47.06 [34.95-79.88]	31.28 [23.23-53.1]	46.36 [28.81-65.41]	25.63 [15.92-36.16]	-0.23 [-0.54 to 0.08]

Australasia	7.47 [6.04-8.06]	16.28 [13.17-17.58]	3.83 [3.13-5]	6.99 [5.72-9.13]	-2.49 [-2.72 to -2.26]
Caribbean	59.73 [49.94-70.69]	52.33 [43.76-61.93]	46.06 [31.81-67.75]	39.42 [27.23-57.99]	-0.53 [-0.73 to -0.33]
Central Asia	76.23 [67.58-93.94]	30.55 [27.08-37.64]	64.25 [38.23-85.15]	23.88 [14.21-31.65]	0.28 [-0.09 to 0.65]
Central Europe	85.81 [65.03-94.56]	29.64 [22.46-32.67]	16.69 [12.81-21.29]	9.46 [7.26-12.07]	-3.49 [-3.64 to -3.35]
Central Latin America	247.71 [206.64-375.45]	38.65 [32.24-58.57]	152.18 [105.21-204.79]	23.27 [16.09-31.32]	-1.55 [-1.67 to -1.42]
Central Sub-Saharan Africa	187.89 [67.25-379.44]	72.73 [26.03-146.88]	175.71 [108.07-282.3]	30.8 [18.94-49.48]	-2.57 [-2.85 to -2.29]
East Asia	1775.41 [1375.32-2410.8]	53.01 [41.06-71.98]	318.89 [262.56-391.93]	13.7 [11.28-16.84]	-4.42 [-4.52 to -4.33]
Eastern Europe	144.12 [103.01-157.28]	28.01 [20.02-30.57]	44.95 [34.76-58.52]	12.13 [9.38-15.79]	-2.07 [-2.54 to -1.6]
Eastern Sub-Saharan Africa	655.54 [228.08-1281.8]	72.54 [25.24-141.84]	632.19 [386.55-1004.01]	35.81 [21.9-56.87]	-2.1 [-2.25 to -1.95]
High-income Asia Pacific	55.27 [45.68-60.14]	15.68 [12.96-17.07]	12.97 [11.61-15.05]	5.56 [4.97-6.45]	-3.61 [-3.82 to -3.4]
High-income North America	110.49 [94.88-117.62]	17.99 [15.45-19.15]	63.38 [56.94-76.31]	9.56 [8.59-11.52]	-1.76 [-1.98 to -1.54]
North Africa and Middle East	1435.37 [849.56-2019.04]	99.87 [59.11-140.48]	671.51 [540.04-816.25]	38.19 [30.71-46.42]	-3.03 [-3.17 to -2.89]
Oceania	12.98 [4.64-21.75]	49.39 [17.67-82.76]	22.86 [8.9-38.77]	47.34 [18.43-80.28]	0.16 [0 to 0.31]

South Asia	1840.02 [1300.38-2468.33]	41.92 [29.62-56.23]	1058.78 [775.12-1445.28]	20.49 [15-27.97]	-2.27 [-2.35 to -2.19]
Southeast Asia	699.77 [388.19-1362.08]	40.63 [22.54-79.08]	371.97 [298.2-493.86]	22.04 [17.67-29.26]	-1.85 [-1.98 to -1.72]
Southern Latin America	49.28 [40.97-58.65]	33.01 [27.44-39.28]	29.21 [19.76-37.72]	19.6 [13.26-25.3]	-1.42 [-1.59 to -1.25]
Southern Sub-Saharan Africa	39.19 [31.1-48.79]	19.23 [15.26-23.95]	41.55 [29.46-55.42]	17.6 [12.48-23.47]	0.34 [0.09 to 0.59]
Tropical Latin America	404.27 [198.55-549.58]	74.94 [36.81-101.88]	164.63 [130.81-204.23]	33.12 [26.31-41.08]	-2.34 [-2.49 to -2.18]
Western Europe	123.72 [100.78-133.24]	17.41 [14.18-18.75]	45.34 [38.21-59.39]	6.59 [5.55-8.63]	-3.05 [-3.22 to -2.87]
Western Sub-Saharan Africa	664.21 [209.26-1448.2]	75.64 [23.83-164.91]	1030 [439.24-1839.28]	51.93 [22.15-92.74]	-1.17 [-1.3 to -1.04]

TableS2: DALYs of congenital birth defects between 1990 and 2019 in 0 to 14 years at the global and regional level

Location	1990		2019		EAPC_95%CI
	Number_95%UI	ASR	Number_95%UI	ASR	
Overall	794401.02 [547839.35-1170085.51]	4529.16 [3123.43-6671.08]	469083.64 [379015.44-601305.28]	2393.61 [1934.02-3068.3]	-1.93 [-2.02 to -1.84]
Female	357417.39 [246871.87-551932]	4188.76 [2893.22-6468.37]	211234.29 [170016.61-271612.94]	2228.09 [1793.33-2864.96]	-1.93 [-2.01 to -1.84]
Male	436983.63 [285232.06-700745.84]	4851.65 [3166.82-7780.1]	257849.35 [202520.81-341103.68]	2548.72 [2001.82-3371.65]	-1.93 [-2.03 to -1.83]
High SDI	33297.15 [30580.43-36884.83]	1928.3 [1770.97-2136.07]	14214.91 [12533.93-16549.52]	870.68 [767.72-1013.68]	-2.53 [-2.68 to -2.39]
High-middle SDI	118001.99 [95597.64-142496.89]	3887.51 [3149.41-4694.48]	35390.09 [30174.48-41639.63]	1443.21 [1230.51-1698.06]	-3.13 [-3.21 to -3.05]
Middle SDI	256734.55 [194216.2-368656.33]	4416.14 [3340.75-6341.32]	100876.61 [83267.34-123042.33]	1822.2 [1504.11-2222.59]	-2.66 [-2.79 to -2.53]

Low-middle SDI	223314.26 [148021.77-327654.31]	4925.35 [3264.72-7226.65]	131570.78 [109050.89-160911.71]	2513.06 [2082.92-3073.48]	-2.08 [-2.17 to -1.99]
Low SDI	162579.38 [69697.77-295831.13]	6717.71 [2879.88-12223.62]	186628 [113288.96-290205.87]	3946.66 [2395.74-6137.04]	-1.72 [-1.8 to -1.64]
Andean Latin America	4314.27 [3244.96-7195.26]	2867.68 [2156.92-4782.67]	4286.83 [2788.65-5945.23]	2369.61 [1541.47-3286.32]	-0.22 [-0.51 to 0.08]
Australasia	720.47 [596.47-778.03]	1570.65 [1300.33-1696.15]	413 [347.47-514.31]	753.16 [633.66-937.93]	-2.16 [-2.37 to -1.95]
Caribbean	5393.94 [4526.88-6332.25]	4725.91 [3966.24-5548.01]	4207.38 [2947.87-6107.97]	3601.08 [2523.08-5227.8]	-0.5 [-0.7 to -0.31]
Central Asia	7095.4 [6378.18-8600.53]	2843.17 [2555.78-3446.29]	6061.03 [3836.34-7949.7]	2253.19 [1426.16-2955.31]	0.28 [-0.07 to 0.63]
Central Europe	7953.31 [6095.04-8743.65]	2747.58 [2105.62-3020.62]	1689.58 [1336.94-2108.23]	958.01 [758.05-1195.38]	-3.24 [-3.37 to -3.1]
Central Latin America	23071.62 [19420.2-34074.15]	3599.46 [3029.79-5316]	14573.46 [10481.37-19161.93]	2228.76 [1602.94-2930.48]	-1.48 [-1.6 to -1.37]
Central Sub-Saharan Africa	17039.85 [6449.37-33868.65]	6596.15 [2496.56-13110.6]	16482.09 [10556.76-25798.86]	2889.03 [1850.42-4522.1]	-2.47 [-2.74 to -2.21]
East Asia	161218.21 [126010.98-217630.1]	4813.36 [3762.21-6497.6]	31370.55 [26197.56-37827.73]	1348.08 [1125.78-1625.56]	-4.18 [-4.27 to -4.09]

Eastern Europe	13447.78 [9842.83-14729.62]	2613.77 [1913.09-2862.91]	4435.94 [3534.87-5664.48]	1196.99 [953.84-1528.49]	-1.91 [-2.35 to -1.47]
Eastern Sub-Saharan Africa	59464.63 [21969.28-114779.89]	6580.31 [2431.1-12701.45]	58667.63 [37331.03-91727.64]	3323.38 [2114.71-5196.15]	-2.04 [-2.18 to -1.9]
High-income Asia Pacific	5618.33 [4755.57-6197.42]	1594.19 [1349.38-1758.51]	1647.86 [1434.42-1895.46]	705.95 [614.51-812.03]	-2.84 [-3.04 to -2.64]
High-income North America	10491.59 [9152.34-11174.44]	1708.39 [1490.32-1819.58]	6363.35 [5719.75-7479.22]	960.2 [863.09-1128.58]	-1.62 [-1.83 to -1.41]
North Africa and Middle East	128959.81 [77203.58-179839.78]	8972.84 [5371.72-12513]	61967.38 [50523.72-74661.97]	3524.02 [2873.23-4245.95]	-2.95 [-3.08 to -2.82]
Oceania	1185.51 [454.28-1956.35]	4511.63 [1728.84-7445.17]	2095.84 [864.06-3492.08]	4339.25 [1788.95-7230.04]	0.16 [0.01 to 0.31]
South Asia	167136.83 [119989.19-222009.42]	3807.39 [2733.36-5057.39]	98380.35 [73694.4-132006.14]	1903.79 [1426.09-2554.5]	-2.19 [-2.27 to -2.12]
Southeast Asia	64511.98 [36746.43-122348.85]	3745.69 [2133.57-7103.81]	35133.4 [28663.98-45687.71]	2081.69 [1698.37-2707.04]	-1.78 [-1.9 to -1.65]
Southern Latin America	4622.89 [3893.78-5479.06]	3096.4 [2608.04-3669.86]	2850.66 [2005.84-3609.27]	1912.41 [1345.65-2421.34]	-1.31 [-1.46 to -1.15]
Southern Sub-Saharan Africa	3806.81 [3070.99-4682.13]	1868.35 [1507.21-2297.95]	4039.04 [2998.57-5259.18]	1710.42 [1269.81-2227.12]	0.33 [0.09 to 0.56]

Tropical Latin America	36312.07 [18176.39-48962.1]	6731.36 [3369.46-9076.37]	15093.67 [12119.14-18676.34]	3036.18 [2437.84-3756.85]	-2.28 [-2.43 to -2.13]
Western Europe	11837.27 [9850.32-12749.58]	1666.01 [1386.36-1794.41]	4910.38 [4189.67-6148.22]	713.49 [608.77-893.35]	-2.66 [-2.84 to -2.49]
Western Sub-Saharan Africa	60198.44 [20502.15-129336.59]	6854.99 [2334.65-14727.97]	94414.2 [42970.06-164976.06]	4760.44 [2166.59-8318.23]	-1.14 [-1.26 to -1.01]

TableS3:The local drift of prevalence from 1990 to 2019 for CBDs disorders across SDI quintiles Location

Location	Age	Local drift (%/year)	95%CI_low	95%CI_high	sex
Global	0 to 4	-0.033872003	-0.06437704	-0.003357654	Male
Global	5 to 9	-0.349257716	-0.384418429	-0.314084592	Male
Global	10 to 14	-0.569484888	-0.622736157	-0.516205084	Male
High SDI	0 to 4	-0.208645588	-0.245055311	-0.172222576	Male
High SDI	5 to 9	-0.264570798	-0.30633612	-0.22278798	Male
High SDI	10 to 14	-0.331753567	-0.393052861	-0.270416548	Male
High-middle SDI	0 to 4	-0.021239177	-0.062529374	0.02006808	Male
High-middle SDI	5 to 9	-0.368522349	-0.414777429	-0.322245784	Male
High-middle SDI	10 to 14	-0.503427224	-0.571840975	-0.434966399	Male
Low-middle SDI	0 to 4	-0.090546887	-0.139952622	-0.041116709	Male
Low-middle SDI	5 to 9	-0.412855606	-0.47097425	-0.354703025	Male
Low-middle SDI	10 to 14	-0.670523043	-0.759246397	-0.581720369	Male
Middle SDI	0 to 4	0.014496096	-0.014082635	0.043082995	Male
Middle SDI	5 to 9	-0.359712435	-0.391115425	-0.328299544	Male
Middle SDI	10 to 14	-0.602805774	-0.649434981	-0.556154682	Male
Low SDI	0 to 4	-0.168018128	-0.178533772	-0.157501376	Male
Low SDI	5 to 9	-0.381029233	-0.394240295	-0.367816418	Male
Low SDI	10 to 14	-0.509851495	-0.531142153	-0.48855628	Male
Global	0 to 4	0.054076631	0.040957892	0.067197091	Female
Global	5 to 9	-0.230464627	-0.245822625	-0.215104263	Female
Global	10 to 14	-0.414700023	-0.438164362	-0.391230153	Female
High SDI	0 to 4	-0.379725874	-0.422606158	-0.336827123	Female

High SDI	5 to 9	-0.328444392	-0.377093049	-0.279771979	Female
High SDI	10 to 14	-0.334614152	-0.406343355	-0.262833288	Female
High-middle SDI	0 to 4	0.106295218	0.082380156	0.130215995	Female
High-middle SDI	5 to 9	-0.262704094	-0.289865166	-0.235535623	Female
High-middle SDI	10 to 14	-0.421202831	-0.461478222	-0.380911144	Female
Low-middle SDI	0 to 4	0.042036789	0.018278082	0.065801139	Female
Low-middle SDI	5 to 9	-0.313925788	-0.342306946	-0.285536548	Female
Low-middle SDI	10 to 14	-0.541100642	-0.58501599	-0.497165894	Female
Middle SDI	0 to 4	0.044510299	0.025514864	0.063509341	Female
Middle SDI	5 to 9	-0.237214373	-0.258468977	-0.21595524	Female
Middle SDI	10 to 14	-0.399253413	-0.43098934	-0.36750737	Female
Low SDI	0 to 4	-0.088617414	-0.10455847	-0.072673813	Female
Low SDI	5 to 9	-0.267268477	-0.287703349	-0.246829418	Female
Low SDI	10 to 14	-0.370005184	-0.403302276	-0.336696959	Female
Global	0 to 4	0.008288112	-0.011188013	0.02776803	Both
Global	5 to 9	-0.293262581	-0.315876358	-0.270643674	Both
Global	10 to 14	-0.496408212	-0.530799208	-0.462005326	Both
High SDI	0 to 4	-0.290135267	-0.32498514	-0.255273209	Both
High SDI	5 to 9	-0.295125843	-0.334893318	-0.255342499	Both
High SDI	10 to 14	-0.333177433	-0.391673529	-0.274646985	Both
High-middle SDI	0 to 4	0.039128867	0.006068157	0.072200507	Both
High-middle SDI	5 to 9	-0.31720863	-0.354482015	-0.279921301	Both
High-middle SDI	10 to 14	-0.461936133	-0.517131906	-0.406709735	Both
Low-middle SDI	0 to 4	-0.027130904	-0.053699543	-0.000555203	Both
Low-middle SDI	5 to 9	-0.367304227	-0.398784354	-0.33581415	Both

Low-middle SDI	10 to 14	-0.611116558	-0.659482122	-0.562727446	Both
Middle SDI	0 to 4	0.029262079	0.006060452	0.052469088	Both
Middle SDI	5 to 9	-0.30135885	-0.327068759	-0.275642309	Both
Middle SDI	10 to 14	-0.505766131	-0.544041137	-0.467476396	Both
Low SDI	0 to 4	-0.128687513	-0.137700083	-0.11967413	Both
Low SDI	5 to 9	-0.326368966	-0.337802904	-0.314933716	Both
Low SDI	10 to 14	-0.442926101	-0.461451911	-0.424396844	Both

TableS4：Age effects on CBDs disorders prevalence across SDI quintiles

Rate(per 100,000 population)	95%CI_low	95%CI_high	location	sex	label
2443.181072	2427.432667	2459.031648	Global	Male	0 to 4
944.1089868	937.0378192	951.2335156	Global	Male	5 to 9
762.6318828	756.8312055	768.477019	Global	Male	10 to 14
2359.362758	2341.146327	2377.720931	High SDI	Male	0 to 4
840.6506135	833.1557203	848.2129291	High SDI	Male	5 to 9
695.7427919	689.4727497	702.0698537	High SDI	Male	10 to 14
2371.439759	2350.719082	2392.343082	High-middle SDI	Male	0 to 4
933.0687513	923.8527324	942.3767059	High-middle SDI	Male	5 to 9
759.8049415	752.4676684	767.21376	High-middle SDI	Male	10 to 14
2403.694812	2378.915163	2428.732575	Low-middle SDI	Male	0 to 4
896.4456099	885.6011236	907.4228906	Low-	Male	5 to 9

			middle SDI		
712.3609598	703.4410633	721.3939639	Low-middle SDI	Male	10 to 14
2353.379867	2339.519535	2367.322313	Middle SDI	Male	0 to 4
975.2723876	968.7504947	981.8381876	Middle SDI	Male	5 to 9
804.8844199	799.5566014	810.2477402	Middle SDI	Male	10 to 14
2684.204045	2678.006785	2690.415647	Low SDI	Male	0 to 4
1011.57388	1008.746205	1014.409482	Low SDI	Male	5 to 9
794.8262213	792.4343803	797.2252817	Low SDI	Male	10 to 14
2342.950409	2336.335762	2349.583783	Global	Female	0 to 4
886.1435344	883.229538	889.0671448	Global	Female	5 to 9
694.967702	692.63006	697.3132335	Global	Female	10 to 14
2282.126803	2261.658361	2302.780489	High SDI	Female	0 to 4
840.7016463	832.0317736	849.4618602	High SDI	Female	5 to 9
679.9871205	672.8734217	687.1760262	High	Female	10 to 14

			SDI		
2274.655135	2263.004829	2286.365418	High-middle SDI	Female	0 to 4
867.8126546	862.7710593	872.8837105	High-middle SDI	Female	5 to 9
694.9446861	690.9949476	698.9170014	High-middle SDI	Female	10 to 14
2251.412156	2239.947467	2262.935525	Low-middle SDI	Female	0 to 4
837.8023466	832.8032079	842.831494	Low-middle SDI	Female	5 to 9
637.529721	633.5501958	641.5342429	Low-middle SDI	Female	10 to 14
2235.219411	2226.353066	2244.121066	Middle SDI	Female	0 to 4
895.6488782	891.5973884	899.7187784	Middle SDI	Female	5 to 9
720.762958	717.5171506	724.0234483	Middle SDI	Female	10 to 14

2655.325871	2645.800181	2664.885855	Low SDI	Female	0 to 4
967.4338515	963.2206095	971.6655229	Low SDI	Female	5 to 9
741.3524269	737.8471936	744.8743123	Low SDI	Female	10 to 14
2394.985728	2385.041166	2404.971753	Global	Both	0 to 4
916.1405117	911.7151407	920.587363	Global	Both	5 to 9
729.8681762	726.2757371	733.4783849	Global	Both	10 to 14
2321.905317	2304.850347	2339.086488	High SDI	Both	0 to 4
840.6693622	833.5530445	847.8464342	High SDI	Both	5 to 9
688.0924463	682.194468	694.0414162	High SDI	Both	10 to 14
2325.045818	2308.688583	2341.518944	High-middle SDI	Both	0 to 4
901.682526	894.5002611	908.9224598	High-middle SDI	Both	5 to 9
728.4711254	722.7963385	734.190466	High-middle SDI	Both	10 to 14
2330.624886	2317.526058	2343.79775	Low-	Both	0 to 4

			middle SDI		
868.2994047	862.5755484	874.0612432	Low-middle SDI	Both	5 to 9
676.2308037	671.593706	680.8999188	Low-middle SDI	Both	10 to 14
2296.768878	2285.71879	2307.872388	Middle SDI	Both	0 to 4
936.9914946	931.8624793	942.1487402	Middle SDI	Both	5 to 9
764.2501819	760.0983256	768.4247168	Middle SDI	Both	10 to 14
2670.514152	2665.163754	2675.875292	Low SDI	Both	0 to 4
990.0324731	987.6275667	992.4432356	Low SDI	Both	5 to 9
768.657442	766.6391446	770.6810529	Low SDI	Both	10 to 14

TableS5：Period effects on CBDs disorders prevalence across SDI quintiles					
Rate Ratio	95%CI_low	95%CI_high	location	sex	label
0.985511126	0.976800721	0.994299203	Global	Male	1990 to 1994
0.992699499	0.984370203	1.001099275	Global	Male	1995 to 1999
1	1	1	Global	Male	2000 to 2004
0.983945419	0.975734411	0.992225525	Global	Male	2005 to 2009
0.951608336	0.943167338	0.960124879	Global	Male	2010 to 2014
0.908970284	0.899435639	0.918606002	Global	Male	2015 to 2019
1.052898958	1.041953538	1.063959357	High SDI	Male	1990 to 1994
1.02549012	1.015278942	1.035803998	High SDI	Male	1995 to 1999
1	1	1	High SDI	Male	2000 to 2004
0.98159069	0.97174364	0.991537525	High SDI	Male	2005 to 2009
0.974633558	0.964304794	0.985072954	High SDI	Male	2010 to 2014
0.990328072	0.977973153	1.002839074	High SDI	Male	2015 to 2019
0.996699059	0.985333116	1.00819611	High-middle SDI	Male	1990 to 1994
0.99154628	0.980672814	1.002540309	High-middle SDI	Male	1995 to 1999
1	1	1	High-middle SDI	Male	2000 to 2004
0.986838118	0.975666173	0.998137987	High-middle SDI	Male	2005 to 2009
0.961224132	0.94967961	0.972908993	High-middle SDI	Male	2010 to 2014
0.922068697	0.909081515	0.935241415	High-middle SDI	Male	2015 to 2019
0.972024446	0.958069452	0.986182706	Low-middle SDI	Male	1990 to 1994
0.984257209	0.970936129	0.997761053	Low-middle SDI	Male	1995 to 1999
1	1	1	Low-middle SDI	Male	2000 to 2004
0.983364132	0.970249038	0.996656506	Low-middle SDI	Male	2005 to 2009
0.934068855	0.920690226	0.947641891	Low-middle SDI	Male	2010 to 2014
0.878101903	0.863051417	0.893414851	Low-middle SDI	Male	2015 to 2019
0.990021918	0.982226148	0.997879563	Middle SDI	Male	1990 to 1994
0.995025476	0.987588207	1.002518753	Middle SDI	Male	1995 to

					1999
1	1	1	Middle SDI	Male	2000 to 2004
0.98030807	0.972869358	0.987803659	Middle SDI	Male	2005 to 2009
0.954105678	0.946425879	0.961847796	Middle SDI	Male	2010 to 2014
0.914940685	0.906307697	0.923655907	Middle SDI	Male	2015 to 2019
0.992563171	0.989193473	0.995944347	Low SDI	Male	1990 to 1994
0.999454229	0.996295023	1.002623454	Low SDI	Male	1995 to 1999
1	1	1	Low SDI	Male	2000 to 2004
0.987769363	0.984825019	0.990722509	Low SDI	Male	2005 to 2009
0.956484445	0.95343419	0.959544458	Low SDI	Male	2010 to 2014
0.905397277	0.901942519	0.908865267	Low SDI	Male	2015 to 2019
0.992394942	0.988550501	0.996254333	Global	Female	1990 to 1994
0.994986272	0.991321124	0.998664972	Global	Female	1995 to 1999
1	1	1	Global	Female	2000 to 2004
0.994783012	0.991144531	0.99843485	Global	Female	2005 to 2009
0.975378328	0.971584062	0.979187411	Global	Female	2010 to 2014
0.94112794	0.936796486	0.945479423	Global	Female	2015 to 2019
1.051182136	1.038464568	1.06405545	High SDI	Female	1990 to 1994
1.025335997	1.013525171	1.037284456	High SDI	Female	1995 to 1999
1	1	1	High SDI	Female	2000 to 2004
0.969835111	0.958575254	0.981227231	High SDI	Female	2005 to 2009
0.953090894	0.941326576	0.965002237	High SDI	Female	2010 to 2014
0.975668425	0.961497216	0.990048499	High SDI	Female	2015 to 2019
0.984235443	0.977654546	0.990860637	High-middle SDI	Female	1990 to 1994
0.987876247	0.981512888	0.994280861	High-middle SDI	Female	1995 to 1999
1	1	1	High-middle SDI	Female	2000 to 2004
1.000636044	0.993980024	1.007336635	High-middle SDI	Female	2005 to 2009
0.98016273	0.973246883	0.98712772	High-middle SDI	Female	2010 to 2014

0.929759704	0.922034662	0.937549469	High-middle SDI	Female	2015 to 2019
0.991867781	0.98484837	0.998937222	Low-middle SDI	Female	1990 to 1994
0.991235753	0.984611463	0.997904611	Low-middle SDI	Female	1995 to 1999
1	1	1	Low-middle SDI	Female	2000 to 2004
0.998395725	0.99183157	1.005003322	Low-middle SDI	Female	2005 to 2009
0.96914367	0.962297733	0.97603831	Low-middle SDI	Female	2010 to 2014
0.919244237	0.911486139	0.927068369	Low-middle SDI	Female	2015 to 2019
0.994173809	0.988896456	0.999479325	Middle SDI	Female	1990 to 1994
0.995210291	0.990183713	1.000262386	Middle SDI	Female	1995 to 1999
1	1	1	Middle SDI	Female	2000 to 2004
0.990608844	0.985534875	0.995708937	Middle SDI	Female	2005 to 2009
0.974462284	0.969160014	0.979793562	Middle SDI	Female	2010 to 2014
0.943919986	0.937881476	0.949997375	Middle SDI	Female	2015 to 2019
1.017129715	1.011777806	1.022509934	Low SDI	Female	1990 to 1994
1.009101507	1.004135719	1.014091852	Low SDI	Female	1995 to 1999
1	1	1	Low SDI	Female	2000 to 2004
0.996956624	0.99233653	1.001598227	Low SDI	Female	2005 to 2009
0.983455225	0.978583978	0.988350719	Low SDI	Female	2010 to 2014
0.952414744	0.946780212	0.95808281	Low SDI	Female	2015 to 2019
0.988552854	0.982922186	0.994215777	Global	Both	1990 to 1994
0.993700417	0.988323058	0.999107033	Global	Both	1995 to 1999
1	1	1	Global	Both	2000 to 2004
0.989041986	0.983722979	0.994389752	Global	Both	2005 to 2009
0.962710363	0.957205558	0.968246826	Global	Both	2010 to 2014
0.923900201	0.917651068	0.930191891	Global	Both	2015 to 2019
1.052012292	1.041597053	1.062531676	High SDI	Both	1990 to 1994
1.025355394	1.015660274	1.035143061	High SDI	Both	1995 to 1999
1	1	1	High SDI	Both	2000 to

					2004
0.975943883	0.966645036	0.985332182	High SDI	Both	2005 to 2009
0.964274069	0.954538031	0.974109412	High SDI	Both	2010 to 2014
0.98324246	0.971556653	0.995068823	High SDI	Both	2015 to 2019
0.990692768	0.981595296	0.999874556	High-middle SDI	Both	1990 to 1994
0.989697712	0.980950571	0.998522852	High-middle SDI	Both	1995 to 1999
1	1	1	High-middle SDI	Both	2000 to 2004
0.993346914	0.984282238	1.002495071	High-middle SDI	Both	2005 to 2009
0.970221718	0.96082968	0.979705562	High-middle SDI	Both	2010 to 2014
0.925933876	0.915402286	0.936586631	High-middle SDI	Both	2015 to 2019
0.980937499	0.97326405	0.988671447	Low-middle SDI	Both	1990 to 1994
0.987394871	0.980107853	0.994736067	Low-middle SDI	Both	1995 to 1999
1	1	1	Low-middle SDI	Both	2000 to 2004
0.990296494	0.98310021	0.997545454	Low-middle SDI	Both	2005 to 2009
0.950133267	0.942716283	0.957608605	Low-middle SDI	Both	2010 to 2014
0.896877562	0.888503258	0.905330796	Low-middle SDI	Both	2015 to 2019
0.991716492	0.985334488	0.998139833	Middle SDI	Both	1990 to 1994
0.995039532	0.988954445	1.00116206	Middle SDI	Both	1995 to 1999
1	1	1	Middle SDI	Both	2000 to 2004
0.9851293	0.97901616	0.991280611	Middle SDI	Both	2005 to 2009
0.963637066	0.957289699	0.970026519	Middle SDI	Both	2010 to 2014
0.928482349	0.921303192	0.935717449	Middle SDI	Both	2015 to 2019
1.004245212	1.001289813	1.007209334	Low SDI	Both	1990 to 1994
1.004003852	1.001246831	1.006768465	Low SDI	Both	1995 to 1999
1	1	1	Low SDI	Both	2000 to 2004
0.992226532	0.989658836	0.994800891	Low SDI	Both	2005 to 2009
0.969385968	0.966703361	0.972076019	Low SDI	Both	2010 to 2014
0.927726398	0.924656918	0.930806068	Low SDI	Both	2015 to 2019

TableS6:	Cohort effects on CBDs disorders prevalence across SDI quintiles					
Cohort	Rate Ratio	95%CI_low	95%CI_high	location	sex	label
1980	1.08418376	1.068241445	1.100363997	Global	Male	1975 to 1984
1985	1.044913987	1.034488612	1.055444427	Global	Male	1980 to 1989
1990	1	1	1	Global	Male	1985 to 1994
1995	0.967406661	0.959172815	0.97571119	Global	Male	1990 to 1999
2000	0.9484806	0.940281819	0.956750871	Global	Male	1995 to 2004
2005	0.947112272	0.939284862	0.955004911	Global	Male	2000 to 2009
2010	0.958902874	0.950951185	0.966921053	Global	Male	2005 to 2014
2015	0.993748644	0.984434164	1.003151256	Global	Male	2010 to 2019
1980	1.055018516	1.037604739	1.072724543	High SDI	Male	1975 to 1984
1985	1.030357669	1.018334456	1.042522836	High SDI	Male	1980 to 1989
1990	1	1	1	High SDI	Male	1985 to 1994
1995	0.992265263	0.982292903	1.002338863	High SDI	Male	1990 to 1999
2000	0.982695602	0.972601143	0.992894829	High SDI	Male	1995 to 2004
2005	0.967753004	0.958232538	0.97736806	High SDI	Male	2000 to 2009
2010	0.959626383	0.95006855	0.969280369	High SDI	Male	2005 to 2014
2015	0.951258084	0.940527536	0.962111057	High SDI	Male	2010 to 2019
1980	1.052514382	1.033577079	1.071798657	High-middle SDI	Male	1975 to 1984
1985	1.034733226	1.021998389	1.047626748	High-middle SDI	Male	1980 to 1989
1990	1	1	1	High-middle SDI	Male	1985 to 1994
1995	0.969373555	0.958750964	0.980113841	High-middle SDI	Male	1990 to 1999
2000	0.945493794	0.934705919	0.956406177	High-middle SDI	Male	1995 to 2004
2005	0.936948852	0.926566915	0.947447116	High-middle SDI	Male	2000 to 2009
2010	0.9502663	0.939587897	0.961066218	High-	Male	2005 to

	77			middle SDI		2014
2015	1.006345896	0.993562983	1.019293271	High-middle SDI	Male	2010 to 2019
1980	1.107029883	1.079815903	1.134929721	Low-middle SDI	Male	1975 to 1984
1985	1.053240641	1.035880236	1.07089199	Low-middle SDI	Male	1980 to 1989
1990	1	1	1	Low-middle SDI	Male	1985 to 1994
1995	0.965057369	0.951705572	0.978596483	Low-middle SDI	Male	1990 to 1999
2000	0.944096577	0.93085347	0.957528091	Low-middle SDI	Male	1995 to 2004
2005	0.940785533	0.928153897	0.953589078	Low-middle SDI	Male	2000 to 2009
2010	0.949413387	0.936588164	0.962414232	Low-middle SDI	Male	2005 to 2014
2015	0.979025913	0.964097733	0.994185244	Low-middle SDI	Male	2010 to 2019
1980	1.084738012	1.070976258	1.098676601	Middle SDI	Male	1975 to 1984
1985	1.047258347	1.038208942	1.056386629	Middle SDI	Male	1980 to 1989
1990	1	1	1	Middle SDI	Male	1985 to 1994
1995	0.960346574	0.953128679	0.96761913	Middle SDI	Male	1990 to 1999
2000	0.94067825	0.933408829	0.948004285	Middle SDI	Male	1995 to 2004
2005	0.943846315	0.93680321	0.950942373	Middle SDI	Male	2000 to 2009
2010	0.959697727	0.952390522	0.967060996	Middle SDI	Male	2005 to 2014
2015	1.004817916	0.996026831	1.013686593	Middle SDI	Male	2010 to 2019
1980	1.059949215	1.053305175	1.066635165	Low SDI	Male	1975 to 1984
1985	1.02814406	1.023926311	1.032379183	Low SDI	Male	1980 to 1989
1990	1	1	1	Low SDI	Male	1985 to 1994
1995	0.972033404	0.968828326	0.975249084	Low SDI	Male	1990 to 1999
2000	0.946896196	0.943828802	0.949973559	Low SDI	Male	1995 to 2004

2005	0.936488715	0.933674837	0.939311074	Low SDI	Male	2000 to 2009
2010	0.940589162	0.937848144	0.94333819	Low SDI	Male	2005 to 2014
2015	0.963757889	0.960651984	0.966873836	Low SDI	Male	2010 to 2019
1980	1.059133163	1.052247321	1.066064066	Global	Female	1975 to 1984
1985	1.02994736	1.025425535	1.034489125	Global	Female	1980 to 1989
1990	1	1	1	Global	Female	1985 to 1994
1995	0.97808533	0.974427291	0.981757101	Global	Female	1990 to 1999
2000	0.960202571	0.956553883	0.963865176	Global	Female	1995 to 2004
2005	0.95935595	0.955887064	0.962837424	Global	Female	2000 to 2009
2010	0.97759322	0.974076384	0.981122754	Global	Female	2005 to 2014
2015	1.019589487	1.015465833	1.023729886	Global	Female	2010 to 2019
1980	1.058405635	1.037997475	1.079215041	High SDI	Female	1975 to 1984
1985	1.027814754	1.013893005	1.041927663	High SDI	Female	1980 to 1989
1990	1	1	1	High SDI	Female	1985 to 1994
1995	1.004366101	0.992667925	1.016202136	High SDI	Female	1990 to 1999
2000	0.990748047	0.978915521	1.002723598	High SDI	Female	1995 to 2004
2005	0.961384339	0.950359092	0.972537491	High SDI	Female	2000 to 2009
2010	0.940493208	0.929557897	0.951557161	High SDI	Female	2005 to 2014
2015	0.916021573	0.90384883	0.928358255	High SDI	Female	2010 to 2019
1980	1.042842704	1.031815898	1.053987352	High-middle SDI	Female	1975 to 1984
1985	1.028021421	1.020594123	1.035502771	High-middle SDI	Female	1980 to 1989
1990	1	1	1	High-middle SDI	Female	1985 to 1994
1995	0.971759518	0.965503485	0.978056088	High-middle SDI	Female	1990 to 1999
2000	0.95085995	0.944479342	0.957283663	High-middle SDI	Female	1995 to 2004
2005	0.948154419	0.941974414	0.954374969	High-middle	Female	2000 to 2009

				SDI		
2010	0.972251894	0.965866457	0.978679546	High-middle SDI	Female	2005 to 2014
2015	1.038159585	1.030491874	1.04588435	High-middle SDI	Female	2010 to 2019
1980	1.067686164	1.054597497	1.080937275	Low-middle SDI	Female	1975 to 1984
1985	1.032046701	1.02364078	1.040521651	Low-middle SDI	Female	1980 to 1989
1990	1	1	1	Low-middle SDI	Female	1985 to 1994
1995	0.968878122	0.962256339	0.975545474	Low-middle SDI	Female	1990 to 1999
2000	0.940600989	0.934073415	0.94717418	Low-middle SDI	Female	1995 to 2004
2005	0.939493647	0.933303313	0.945725039	Low-middle SDI	Female	2000 to 2009
2010	0.965479476	0.95915889	0.971841713	Low-middle SDI	Female	2005 to 2014
2015	1.017200013	1.009721149	1.024734272	Low-middle SDI	Female	2010 to 2019
1980	1.056728642	1.047624486	1.065911915	Middle SDI	Female	1975 to 1984
1985	1.030690821	1.024653691	1.036763521	Middle SDI	Female	1980 to 1989
1990	1	1	1	Middle SDI	Female	1985 to 1994
1995	0.978153886	0.973183197	0.983149963	Middle SDI	Female	1990 to 1999
2000	0.963457952	0.958420338	0.968522044	Middle SDI	Female	1995 to 2004
2005	0.960838091	0.955992438	0.965708305	Middle SDI	Female	2000 to 2009
2010	0.974435322	0.969448594	0.979447702	Middle SDI	Female	2005 to 2014
2015	1.018575457	1.012627598	1.024558251	Middle SDI	Female	2010 to 2019
1980	1.0413074	1.031108943	1.051606727	Low SDI	Female	1975 to 1984
1985	1.01833715	1.011845481	1.024870467	Low SDI	Female	1980 to 1989
1990	1	1	1	Low SDI	Female	1985 to 1994
1995	0.979428275	0.974395107	0.984487441	Low SDI	Female	1990 to 1999

2000	0.958643282	0.953805234	0.96350587	Low SDI	Female	1995 to 2004
2005	0.952311284	0.947892076	0.956751095	Low SDI	Female	2000 to 2009
2010	0.958975109	0.954700914	0.96326844	Low SDI	Female	2005 to 2014
2015	0.983102298	0.978291207	0.987937049	Low SDI	Female	2010 to 2019
1980	1.072101175	1.061897268	1.082403132	Global	Both	1975 to 1984
1985	1.037795351	1.031109592	1.04452446	Global	Both	1980 to 1989
1990	1	1	1	Global	Both	1985 to 1994
1995	0.972367756	0.967027883	0.977737115	Global	Both	1990 to 1999
2000	0.953884543	0.948563399	0.959235536	Global	Both	1995 to 2004
2005	0.952786797	0.947716536	0.957884183	Global	Both	2000 to 2009
2010	0.967695956	0.962549567	0.972869862	Global	Both	2005 to 2014
2015	1.006038859	1.000006535	1.012107571	Global	Both	2010 to 2019
1980	1.05663069	1.039984119	1.073543715	High SDI	Both	1975 to 1984
1985	1.029116364	1.017693053	1.040667898	High SDI	Both	1980 to 1989
1990	1	1	1	High SDI	Both	1985 to 1994
1995	0.99807686	0.988543366	1.007702296	High SDI	Both	1990 to 1999
2000	0.986522477	0.97687606	0.996264151	High SDI	Both	1995 to 2004
2005	0.964664675	0.955619771	0.973795189	High SDI	Both	2000 to 2009
2010	0.950437795	0.941409398	0.959552777	High SDI	Both	2005 to 2014
2015	0.934382602	0.924285749	0.944589753	High SDI	Both	2010 to 2019
1980	1.047439955	1.032247387	1.062856126	High-middle SDI	Both	1975 to 1984
1985	1.03132907	1.021102018	1.041658554	High-middle SDI	Both	1980 to 1989
1990	1	1	1	High-middle SDI	Both	1985 to 1994
1995	0.970653896	0.962080634	0.979303556	High-middle SDI	Both	1990 to 1999
2000	0.94818942	0.939465164	0.956994693	High-middle SDI	Both	1995 to 2004

2005	0.942408428	0.933986921	0.950905871	High-middle SDI	Both	2000 to 2009
2010	0.960714401	0.952033893	0.969474057	High-middle SDI	Both	2005 to 2014
2015	1.021315197	1.010908915	1.0318286	High-middle SDI	Both	2010 to 2019
1980	1.08827172	1.073598915	1.103145058	Low-middle SDI	Both	1975 to 1984
1985	1.043362269	1.033978249	1.052831454	Low-middle SDI	Both	1980 to 1989
1990	1	1	1	Low-middle SDI	Both	1985 to 1994
1995	0.966623234	0.959329178	0.97397275	Low-middle SDI	Both	1990 to 1999
2000	0.942158292	0.934945028	0.949427208	Low-middle SDI	Both	1995 to 2004
2005	0.939931182	0.933069582	0.94684324	Low-middle SDI	Both	2000 to 2009
2010	0.956907607	0.949920884	0.963945717	Low-middle SDI	Both	2005 to 2014
2015	0.99704178	0.988842924	1.005308614	Low-middle SDI	Both	2010 to 2019
1980	1.071131683	1.059987099	1.082393439	Middle SDI	Both	1975 to 1984
1985	1.039347825	1.031988931	1.046759195	Middle SDI	Both	1980 to 1989
1990	1	1	1	Middle SDI	Both	1985 to 1994
1995	0.968659954	0.962701788	0.974654996	Middle SDI	Both	1990 to 1999
2000	0.951333565	0.94531504	0.957390407	Middle SDI	Both	1995 to 2004
2005	0.951902304	0.946090274	0.957750039	Middle SDI	Both	2000 to 2009
2010	0.966710774	0.960703463	0.972755649	Middle SDI	Both	2005 to 2014
2015	1.01139368	1.004195218	1.018643744	Middle SDI	Both	2010 to 2019
1980	1.050755803	1.045020401	1.056522682	Low SDI	Both	1975 to 1984
1985	1.023321641	1.019677117	1.026979192	Low SDI	Both	1980 to 1989
1990	1	1	1	Low SDI	Both	1985 to 1994

1995	0.975485464	0.972689648	0.978289317	Low SDI	Both	1990 to 1999
2000	0.952317279	0.949636363	0.955005763	Low SDI	Both	1995 to 2004
2005	0.94388756	0.941433366	0.946348152	Low SDI	Both	2000 to 2009
2010	0.949418333	0.947035518	0.951807143	Low SDI	Both	2005 to 2014
2015	0.973322358	0.970630243	0.97602194	Low SDI	Both	2010 to 2019
